# Giant Retroperitoneal Lipoma in an Infant

**Published:** 2014-09-01

**Authors:** Manoj Saha

**Affiliations:** Department of Pediatric Surgery, Gauhati Medical College, Guwahati, India

**Keywords:** Retroperitoneal lipomas, Liposarcoma, Infant

## Abstract

Lipomas can occur almost anywhere in the body, but retroperitoneal lipomas are extremely rare. They are slowly growing benign tumors and can attain an enormous size due to silent course of the disease. Total excision of the mass is the treatment of choice and is curative for benign retroperitoneal lipomas. We treated an 11-month-old female patient with giant retroperitoneal lipomas by surgical excision. Histopathology confirmed it as fibrolipoma.

## CASE REPORT

An 11-month-old female child presented with gradual enlargement of the abdomen for the last 8 months, associated with early satiety and constipation. There was no history of vomiting and fever. On examination, her abdomen was distended. Digital rectal examination did not reveal any abnormality. Plain skiagram of the abdomen showed scanty bowel gas. Ultrasonography showed large well-defined multilobulated intra-abdominal soft tissue mass, while liver, spleen and kidneys were seen separately. CT scan showed a large lipomatous mass almost occupying the whole abdomen [Fig. 1]. After preoperative work ups exploration was done through a transverse supra-umbilical incision. There was a large lipomatous mass occupying the retroperitoneum stretching the mesentery and displacing the intestinal loops to the flanks. There was fairly well defined plane of dissection and the mass was excised in toto preserving intestinal circulation [Fig. 2]. There were few enlarged mesenteric lymph nodes and lymph node sampling was also done. Abdomen was closed without drain. The child recovered uneventfully.

**Figure:**
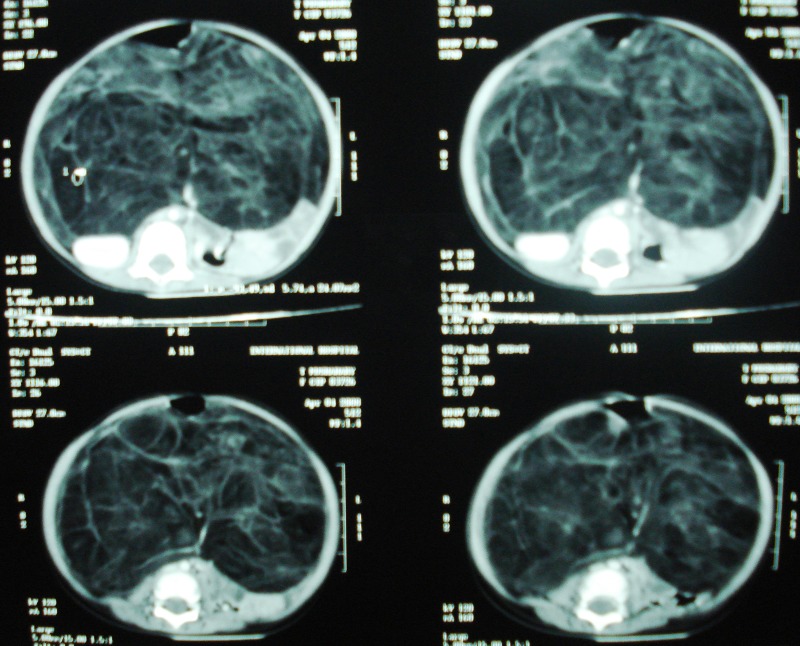
Figure 1:CT scan showing retroperitoneal mass, kidneys are seen separately

**Figure:**
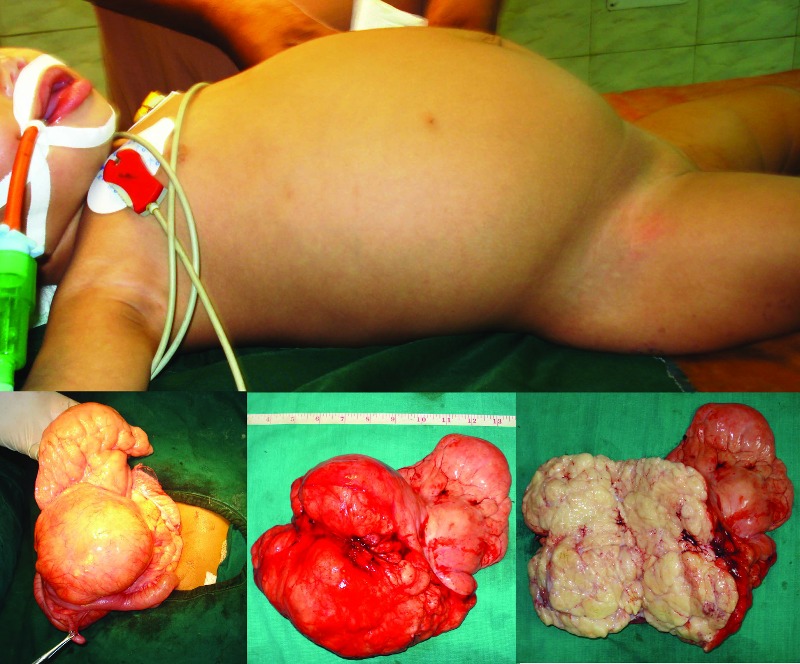
Figure 2:Clinical picture, operative picture and excised specimen- split open

Histopathology of the tumor showed fibrolipoma. Child has been doing well during the five years post-operative follow-up period.

## DISCUSSION

Lipomas are sub-classified into conventional lipoma, fibrolipoma, angiolipoma, fusiform cell lipoma, myolipoma and pleomorphic lipoma [1-3]. Retroperitoneal lipomas are extremely rare, slowly growing benign tumor and few cases have been reported. Although retroperitoneal lipomas are relatively common in adults, they can occur in infant and small children also [3]. They may affect both sexes, but there is greater preponderance for females. Our case is also a female child.

Retroperitoneal lipomas attain considerable dimension, generally presenting diameter greater than 15 cm. Ersue et al reported two cases with dimensions of 15cmsx40cms and 13cmsx18 cms respectively.[4] Jaykar et al also described a similar case with larger dimensions of 40cmsx30cmsx10 cms and weighed 8 kg.[5] Dimensions of our case were 30cmsx25 cms which compares well with the previously reported cases.

CT and MRI are the imaging studies of choice for retroperitoneal lipomas but ultrasonography is ideal forfollow up of these cases. Lipomas have CT signal characteristics similar to that of subcutaneous fat.[1] MRI reveals an intense signal on T1-weighted images.[6] Previously used angiography for lipoma showed the tumor to be hypovascular.[6] 

The treatment of retroperitoneal lipomas is essentially surgical and total excision is the treatment of choice. In almost all the cases surgical resection is feasible because the tumor is well capsulated and always there is clear cleavage between the lipoma and the surrounding structures.

Microscopically, lipoma consists of multivacuolated, small eosinophilic cells and univacuolated adipocytes.[7] Retroperitoneal lipomas should be distinguished from well differentiated liposarcoma of low grade malignancy in order to provide appropriate treatment and follow up. In patients suspected of having retroperitoneal lipomas, a radical resection of the lesion should always be performed if possible, thus reducing the possibility of loco-regional recurrence if the histopathology demonstrates the presence of liposarcoma.

## Footnotes

**Source of Support:** Nil

**Conflict of Interest:** None declared

